# Adoptive immunotherapy of cancer with polyclonal, 10^8^-fold hyperexpanded, CD4^+ ^and CD8^+ ^T cells

**DOI:** 10.1186/1479-5876-2-41

**Published:** 2004-11-26

**Authors:** Li-Xin Wang, Wen-Xin Huang, Hallie Graor, Peter A Cohen, Julian A Kim, Suyu Shu, Gregory E Plautz

**Affiliations:** 1 Center for Surgery Research, The Cleveland Clinic Foundation, Cleveland, OH, USA; 2 Dept. of General Surgery, The Cleveland Clinic Foundation, Cleveland, OH, USA

## Abstract

T cell-mediated cancer immunotherapy is dose dependent and optimally requires participation of antigen-specific CD4^+ ^and CD8^+ ^T cells. Here, we isolated tumor-sensitized T cells and activated them *in vitro *using conditions that led to greater than 10^8^-fold numerical hyperexpansion of either the CD4^+ ^or CD8^+ ^subset while retaining their capacity for *in vivo *therapeutic efficacy. Murine tumor-draining lymph node (TDLN) cells were segregated to purify the CD62L^low ^subset, or the CD4^+ ^subset thereof. Cells were then propagated through multiple cycles of anti-CD3 activation with IL-2 + IL-7 for the CD8^+ ^subset, or IL-7 + IL-23 for the CD4^+ ^subset. A broad repertoire of TCR Vβ families was maintained throughout hyperexpansion, which was similar to the starting population. Adoptive transfer of hyper-expanded CD8^+ ^T cells eliminated established pulmonary metastases, in an immunologically specific fashion without the requirement for adjunct IL-2. Hyper-expanded CD4^+ ^T cells cured established tumors in intracranial or subcutaneous sites that were not susceptible to CD8^+ ^T cells alone. Because accessibility and antigen presentation within metastases varies according to anatomic site, maintenance of a broad repertoire of both CD4^+ ^and CD8^+ ^T effector cells will augment the overall systemic efficacy of adoptive immunotherapy.

## Introduction

Cancer immunotherapy, using T lymphocytes that recognize tumor-specific antigens, holds great promise. Advantageous features include: exquisite specificity for targeted antigens, thereby sparing normal tissues, and the ability of effector T cells to traffic to tumor in all anatomic locations. Although most effector T cells are subject to activation-induced cell death (AICD), a memory response is established leading to sustained protection [1]. Despite the theoretical appeal of T cell-mediated immunotherapy, clinically relevant benefits have been documented in only a small subset of human cancer patients who present with metastatic disease [2-5]. Several factors contribute to the poor host immune response including defective Antigen Presenting Cell (APC) function in cancer patients, and production of immunosuppressive substances by tumors [6-8].

Cognizant of these features, many preclinical studies of active immunotherapy have used a vaccination/challenge scheme to avoid tumor-induced immunosuppression or have alternatively treated hosts with minimal tumor burdens. Several human clinical trials have similarly focused on hosts with minimal residual disease in order to define the magnitude and characteristics of the immune response. These studies have clearly established that immune responses are successfully generated in vaccinated cancer patients. However, the frequency of responding T cells is typically less than one percent even after multiple cycles of vaccination [9-14]. In contrast, the immune response to pathogens generates a tremendous amplification of reactive T cells [15-17]. In the clinical setting, relatively little is yet known about the magnitude of proliferation of individual precursor cells (burst size) as they mature into effector cells, or the flux between lymphoid tissue, peripheral blood, and tumor sites. This results in ambiguity about the optimal time and site to quantify the immune response. Likewise, analysis of apoptosis of effector cells is likely to be important [18,19]. These gaps in fundamental knowledge have made it difficult to identify components of active immunotherapy that could be enhanced to boost the aggregate immune response to a therapeutic level.

Adoptive immunotherapy is another approach to cancer immunotherapy that circumvents some of the limitations of active immunotherapy. Animal tumor models have convincingly demonstrated that hosts bearing progressively growing weakly immunogenic tumors nevertheless generate sensitized T cells in TDLN [20]. Antigen-sensitization causes T cells to downregulate expression of L-selectin (CD62L) providing a convenient phenotypic marker for segregation of primed T cells from the majority of irrelevant T cells [21-23]. Our previous studies have demonstrated that *ex vivo *activation of purified CD62L^low ^T cells from TDLNs generates potent effector CD4^+ ^and CD8^+ ^T cells that can mediate regression of advanced tumors in every tested anatomic location [24-26]. The high potency of such cells permitted brief 5-day activation and limited numerical amplification (10-fold) to supply sufficient quantities of cells for the previous mechanistic analysis of the anti-tumor response. Importantly, these experiments demonstrated that there is tight dose dependence, oftentimes with even a three-fold reduction in the number of transferred cells accounting for a difference between minimal treatment effect and complete cure. The relative efficacy of CD4^+ ^versus CD8^+ ^effector cells also varies considerably between pulmonary metastases and intracranial (i.c.) or subcutaneous (s.c.) tumors [27]. This indicates that maintenance of CD4^+ ^as well as CD8^+ ^tumor-reactive effector T cells would be required for optimal adoptive immunotherapy against disseminated metastatic disease.

We investigated whether we could overcome quantitative limitations associated with active immunotherapy through extensive numerical expansion of effector cells. In a previous study, we determined that *in vitro *activation of tumor-sensitized L-selectin^low ^precursors with anti-CD3 mAb and high concentrations of IL-2 (100 U/ml) induced rapid proliferation of CD8^+ ^effector cells [28]. Adoptive transfer of such cells cured established tumors in recipients. However, these culture conditions led to maximal proliferation in 9 days with subsequent decline in cell numbers thus limiting the total expansion to approximately 10^3^-fold. In this report, we define *ex vivo *activation conditions that permit numerical expansion of either CD4^+ ^or CD8^+ ^effector T cells to greater than 10^8^-fold while retaining their high therapeutic potency and preserving a broad T cell receptor (TCR) repertoire.

## Materials and methods

### Mice and tumors

Female C57BL6N (B6) mice were purchased from the biologic Testing Branch, National Cancer Institute (Frederick, MD). They were maintained in a specific pathogen-free environment according to National Institute of Health guidelines. Mice were used for experiments at 8–10 weeks of age. The MCA 205 and MCA 207 fibrosarcomas, syngeneic to B6 mice were serially passaged in vivo s.c. as described previously [29].

### Preparation and culture activation of TDLN CD62L^low ^cells

Tumors were established by s.c. flank inoculation of 1.5 × 10^6 ^MCA 205 cells and 12 days later the TDLNs were removed and mechanically disrupted to obtain a single cell suspension. The TDLN cells were incubated with 100 μl anti-CD62L microbeads per 10^8 ^cells and applied to MACS columns (Miltenyi Biotech, Auburn CA) and the flow through fraction was collected. For CD4^+ ^hyperexpansion, the CD62L^low ^subset was depleted of CD8^+ ^cells by MACS on day 0 and day 36 of culture activation. CD62L^low ^cells, containing approximately 50% TCR^+ ^and 50% B220^+ ^subsets, were suspended in complete medium (CM) and incubated for 2 days at 4 × 10^6 ^per well in 24 well culture plates coated with anti-CD3 (145-2C11) as previously described [28]. Activated cells were washed, counted, and suspended at 0.5 × 10^5^/ml in CM with IL-2 (4 U/ml) (Chiron Corp. Emeryville, CA), with or without rmIL-7 (10 ng/ml) or rhIL-23 (2 ng/ml) (each from R&D Systems, Minneapolis, MN) and then diluted to 10^5^/ml on day 5 of activation. On days 9 and 15, the cell concentration was adjusted to 2 × 10^5^/ml. For experiments with two cycles of anti-CD3 stimulation, T cells were incubated with immobilized anti-CD3 for 14 hrs on day 15 and used for adoptive therapy on day 23. For long-term expansion, cultures were maintained for 23 days after the initial anti-CD3 stimulation in CM with the indicated combination of IL-2 (4 U/ml), IL-7 (10 ng/ml), and IL-23 (2 ng/ml) and then were stimulated with anti-CD3 for 14 hrs on day 23 and every 7 days thereafter.

### IFN-γ and FACS analysis

T cells were stimulated with a single cell suspension of either MCA 205 or MCA 207 tumors at a 1:1 ratio, or with immobilized anti-CD3. Brefeldin A was added after five hours of stimulation and the cells were harvested after 20 hrs and stained for intracellular IFN-γ according to the manufacturers instructions (BD Biosciences, San Diego, CA). FACS analysis was performed using FITC or PE conjugated antibodies or isotype control antibodies (BD Biosciences).

### RNA isolation and CDR3 size distribution analysis (TCR spectratyping)

TDLN cells were lysed using TRIzol reagent (Invitrogen, Carlsbad, CA) and total RNA was reverse transcribed into cDNA using the SuperScript II RT kit (Invitrogen). cDNA was amplified using PCR with 22 different VB-specific primers paired with a hex-labeled constant region primer which spans the CDR3 region as previously described [30]. CDR3 size distribution analysis was performed by mixing 1.0 μl of hex-labeled PCR amplified cDNAs with 12.0 μl deionized formamide (Sigma) and 0.5 μl size standard (Genescan-400 ROX, ABI 310; Perkin-Elmer, Shelton, CT), heated for 2 minutes at 90°C and chilled on ice prior to analysis. Samples were applied to an ABI 310 sequencer for CDR3 size distribution analysis. Samples were determined to be oligoclonally skewed if the CDR3 size patterns failed to exhibit a Gaussian bell-shaped distribution and were dominated by one or two prominent peaks.

### Adoptive immunotherapy

Mice were inoculated with MCA 205 or MCA 207 tumor cells (3 × 10^5^) i.v. to establish pulmonary metastases. Subcutaneous tumors were established by inoculation of 1.5 × 10^6 ^cells. Intracranial tumors were established by transcranial inoculation of 10^5 ^tumor cells at a depth of 4 mm as previously described [31]. Mice bearing 3-day s.c. or i.c. tumors or 10-day pulmonary metastases were treated with 5 Gy nonmyeloablative total body irradiation (TBI) delivered from a ^137^Cs irradiator prior to intravenous transfer of the T cells whereas mice with 3-day pulmonary tumors were not irradiated. For pulmonary tumors, mice were euthanized on day 20 post inoculation, the lungs were insufflated with India ink and the number of surface tumor nodules was enumerated using a dissecting microscope. Subcutaneous tumors were measured in two perpendicular dimensions three times per week and mice with progressive tumors were euthanized when the product of dimensions exceeded 200 mm^2^. Mice bearing intracranial tumors were monitored daily for survival or were euthanized when neurologic symptoms such as decreased grooming and decreased spontaneous movement were apparent.

### Statistical analysis

Treatment groups consisted of five individuals. Analysis of tumor size for s.c. tumors was performed by the Mann-Whitney rank sum test. For pulmonary tumors, a t test was performed on paired samples and p < 0.05 was considered significant. Survival of mice bearing i.c. tumors was compared using the Wilcoxon rank sum test.

## Results

### Ex vivo stimulation with anti-CD3, IL-2, and IL-7 augments effector cell generation

**Figure 1:**
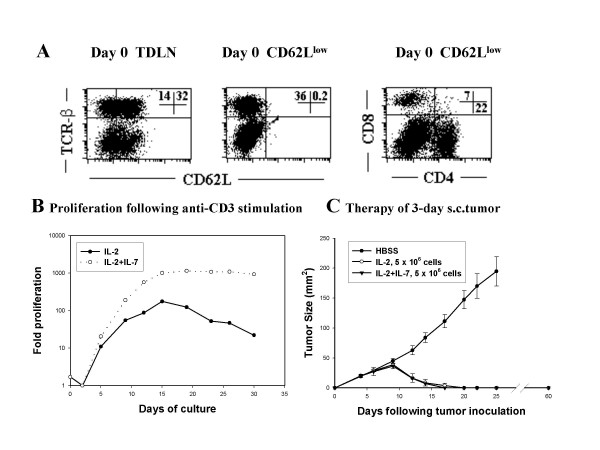
**Proliferation and efficacy of CD62L^low ^TDLN cells cultured with IL-2 +/- IL-7. **(A) Freshly isolated whole TDLN cells were stained for expression of TCR and CD62L (left panel). The purified CD62L^low ^subset was stained for TCR and CD62L expression (center panel), or for CD4 and CD8 expression (right panel). (B) CD62L^low ^TDLN cells were activated with immobilized anti-CD3 mAb for 2 days then cultured in medium with IL-2 (4 U/ml) (closed circle) or the combination of IL-2 and IL-7 (10 ng/ml) (open circle). Cells density was adjusted to 10^5^/ml on days 5 and 9 and total proliferation was calculated. (C) Mice bearing 3-day s.c tumors were treated with 5 Gy TBI then received adoptive transfer of 5 × 10^6 ^T cells cultured for 9 days with IL-2 alone (open circle), IL-2 + IL-7 (open triangle), or HBSS (closed circle). Each treatment group is significantly different than HBSS control (*P *< 0.01).

During the progressive growth of weakly immunogenic tumors an immune response, albeit sub-therapeutic, is initiated in TDLNs. In previous studies our laboratory has demonstrated that the CD62L^low ^subset of T cells contains the tumor-reactive subset whereas the reciprocal CD62L^high ^subset does not have any therapeutic effect and displays suppressor activity [1,24,32]. This finding is consistent with numerous studies documenting the high expression of CD62L on naïve T lymphocytes and its rapid downregulation upon antigen stimulation [21-23]. TDLNs were harvested from mice bearing 12-day subcutaneous MCA 205 tumors and the CD62L^low ^subset was purified by MACS depletion of CD62L^high ^cells. The typical yield of CD62L^low ^cells was 1.5 × 10^6 ^per TDLN, which represented 7–8.5% of the initial cells. The phenotype of the total TDLN prior to MACS separation and the negatively selected CD62L^low ^subset is demonstrated in Figure 1A. The separated cells were highly enriched for the CD62L^low ^fraction consisting of 36% TCR^+ ^cells among which 7% were CD8^+ ^and 22% were CD4^+^. The cells were activated with immobilized anti-CD3 mAb for 48 hrs during which time aggregates of lymphoblasts developed. The activated cells were resuspended at a low density, 0.5 × 10^5^/ml, in medium supplemented with IL-2 (4 U/ml) with or without IL-7 (10 ng/ml) and the cell concentration was adjusted to 10^5^/ml on day 5 and 2 × 10^5^/ml on day 9. As demonstrated in figure 1B, there was an initial 2-fold decline in cell number during the first 48 hrs of culture, primarily though depletion of CD62L^low ^B220^+ ^cells. This was followed by a rapid burst of proliferation from day 2 until day 15 when the IL-2 supplemented culture peaked at 175-fold proliferation and the IL-2 + IL-7 cultures reached 1000-fold proliferation.

The morphology of the cells changed from lymphoblastoid to small round cells at day 15 and there was no additional proliferation. IL-7 preserved the viability of cells whereas IL-2 alone could not prevent a 8-fold numerical decline between days 15 to 30. Because the TDLN cells were initially segregated based on phenotype rather than antigen specificity and the anti-CD3 stimulation was antigen-independent, it was not known whether the enhanced proliferation achieved in the presence of IL-7 was due to preferential growth of irrelevant T cells or preservation of tumor-reactive T cells. As demonstrated in figure 1C, there was equivalent therapeutic efficacy against s.c. tumors at day 9 using T cells cultured with IL-2 alone or the combination of IL-2 and IL-7. Regression of established tumors requires efficient trafficking and the dose of 5 × 10^6 ^CD62L^low ^TDLN cells is near the lowest threshold dose required to cure 3-day s.c. MCA 205 tumors [26,33]. Thus, the addition of IL-7 during *in vitro *activation augmented the total number of cells but did not substantially diminish per-cell therapeutic efficacy.

### Preservation of effector function after anti-CD3 re-stimulation

**Figure 2:**
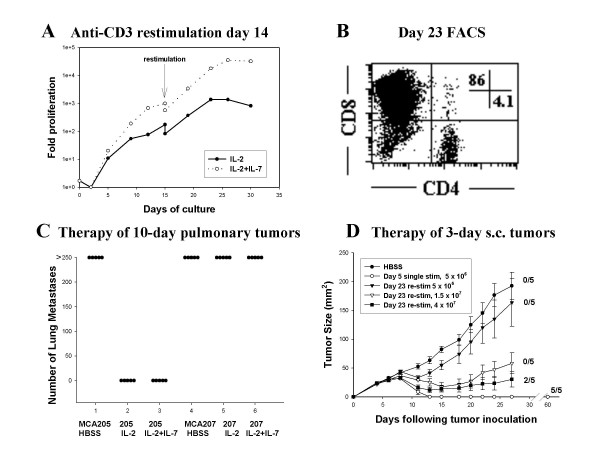
**Restimulation of activated T cells induces additional proliferation with retention of specific anti-tumor efficacy. **(A) CD62L^low ^TDLN cells were activated with anti-CD3 mAb from day 0–2 and again for 14 hrs on day 15. T cells were cultured in the presence of IL-2 (4 U/ml) (closed circle) or IL-2 plus IL-7 (10 ng/ml) (open circle) and the total proliferation is indicated. (B) FACS analysis of activated T cells on day 23 of culture stained for CD4 and CD8. (C) Mice bearing 10-day pulmonary metastases of either MCA205 or MCA207 tumors were pre-treated with 5 Gy TBI then received adoptive transfer of 2.5 × 10^7 ^cells cultured with IL-2 alone, the combination of IL-2 plus IL-7, or control HBSS as indicated. Difference between the groups bearing MCA 205 treated with T cells cultured with IL-2 or IL-2 plus IL-7 and all other groups is (*P *< 0.01). (D) Mice bearing 3-day s.c tumors were pre-treated with 5Gy TBI followed by injection of; HBSS (closed circles), 5 × 10^6 ^T cells activated for 5 days (open circles, *P *< 0.01), 5 × 10^6 ^restimulated T cells at day 23 of culture (closed triangles, *P *= 0.4), 1.5 × 10^7 ^re-stimulated T cells (open triangle, *P *< 0.01), or 4 × 10^7 ^re-stimulated T cells (closed square, *P *< 0.01). Number of mice showing complete regression in each treatment group of 5 mice is indicated in parentheses.

The activated T cells were re-stimulated with anti-CD3 at the time of maximal proliferation on day 15 to determine whether additional numerical expansion could be initiated. Re-stimulated cells rapidly regained lymphoblast morphology and as anticipated nearly half of the cells underwent AICD [34]. The surviving cells underwent a 100-fold numerical expansion before achieving a growth plateau (Figure 2A). In addition, the composition of the T cell cultures changed over time due to the more rapid intrinsic proliferative response of CD8^+ ^T cells [35]. Although CD4^+ ^and CD8^+ ^T cells each proliferated in the presence of IL-2 and IL-7, by day 23 CD8^+ ^cells comprised 86% of the culture whereas there were only 4% CD4^+ ^cells (Figure 2B). The re-stimulated cells cultured in IL-2 or the combination of IL-2 plus IL-7 each retained potent therapeutic efficacy against 10-day MCA 205 pulmonary metastases but not against MCA207, demonstrating retention of antigenic specificity (Figure 2C). The hosts bearing 10-day pulmonary tumors were treated with 5 Gy TBI, which causes transient lymphopenia, prior to adoptive transfer. Moreover, hosts did not receive adjunctive IL-2. Thus, the transferred cells were able to function independently of radiosensitive host cells and were not dependent on exogenous cytokine support. Because the combination of IL-2 plus IL-7 promoted greater numerical expansion of CD8^+ ^T cells with preservation of effector function, it was used for subsequent hyperexpansion studies.

The relative per-cell potency of re-stimulated cultures on day 23 was compared with the 5-day culture activation approach we have employed in previous studies. The segregated CD62L^low ^TDLN cells were frozen and one aliquot was thawed and activated for a total of 23 days with anti-CD3 stimulation on days 0–2 and again on day 15. The second aliquot was thawed on culture day 18 of the first aliquot and stimulated with anti-CD3 for 48 hrs and then cultured with IL-2 and IL-7 for an additional 3 days. The two T cell cultures were synchronously harvested and transferred into hosts bearing 3-day s.c. tumors. As demonstrated in Figure 2D, whereas 5 × 10^6 ^cells activated for 5 days was curative in 5/5 mice, 5 × 10^6 ^cells cultured for 23 days had minimal therapeutic effect. However, a modest increase in the cell dose to 1.5 × 10^7 ^cells led to a significant therapeutic effect and at a dose of 4 × 10^7 ^cells 2/5 mice were cured. The s.c. tumor model is highly dependent on the presence of tumor-specific CD4^+ ^T cells [36,37]. The relative decrease in percentage of CD4^+ ^cells from 24% on day 5 of culture to 4% on day 23 may account for some of the differential therapeutic effects. In contrast to the modest difference in per-cell efficacy, there was nearly 1000-fold greater proliferation in the 23-day versus 5-day cultures indicating that the aggregate therapeutic effect was substantially greater following extended culture.

### Repetitive anti-CD3 stimulation induces hyper-expansion of CD8^+ ^effectors

There are immunologic scenarios that demonstrate exhaustion of the effector response leading to failure of immunologic control of infection or tumor [38-40]. By contrast, selection and extensive propagation of T cell clones indicates that T cells can undergo massive proliferation yet retain antigen-specific function. To assess whether there is an intrinsic limit to the retention of *in vivo *effector function of CD62L^low ^cells, they were stimulated with anti-CD3 followed by IL-2 and IL-7 for 23 days. Starting on day 23, the T cells were activated with anti-CD3 every 7 days. The time course between the initial and subsequent anti-CD3 stimulations was chosen based on evidence that T cells undergo changes in gene expression, phenotype, and function over a twenty-day time course in the transition from naïve to memory cells [41].

**Figure 3:**
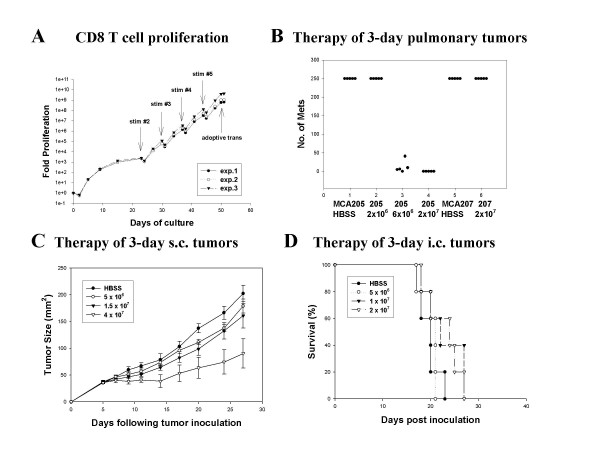
**CD8^+ ^effector T cells can be hyperexpanded through repetitive anti-CD3 stimulation. **(A) CD62L^low ^TDLN cells were restimulated with anti-CD3 mAb for 14 hrs every 7 days starting on day 23 of culture and overall proliferation was measured for three independent experiments. (B) T cells were harvested on day 50 of culture and adoptively transferred to hosts bearing either MCA 205 or MCA 207 3-day pulmonary metastases (*P *< 0.01 for MCA 205 tumors treated with either 6 × 10^6 ^or 2 × 10^7 ^compared with all other groups). (C) Mice bearing 3-day s.c tumors were treated with 5 Gy TBI then received adoptive transfer of the indicated number of T cells hyperexpanded for 50 days (*P *= 0.06 for 4 × 10^7 ^cell dose) (D) Mice bearing 3-day i.c. tumors were treated with 5Gy TBI then received adoptive transfer of the indicated number of T cells hyperexpanded for 50 days. Mice were sacrificed when they developed neurologic symptoms indicating progressive tumor (*P *= 0.9 for treatment groups versus control).

As demonstrated in Figure 3A, repetitive anti-CD3 stimulation was accompanied by immediate AICD in approximately 50% of the cells followed by rapid proliferation. There was no evidence that the T cells became effete over the 50-day expansion period despite a total proliferation of 2–6 × 10^8^-fold that was consistent in three independent experiments. TCR Vβ expression was determined for freshly harvested TDLN T cells, the CD62L^low ^subset, and activated cultures from several independent experiments (Table 1) "see additional file 1". As demonstrated, multiple TCR Vβ families are represented prior to and following hyperexpansion. There is a relatively low level of variability in the prevalence of multiple TCR Vβ families between experiments, especially considering that greater than 10^8^-fold total proliferation had occurred. In addition, TCR spectratype analysis for each Vβ family revealed a poyclonal rather than clonal or oligoclonal distribution (data not shown).

At day 50, the cultures were >99% TCR^+ ^and CD8^+ ^indicating preferential expansion or survival of CD8^+ ^cells under the conditions employed. As shown in Figure 3B, adoptive transfer of 2 × 10^7 ^cells to hosts with 3-day MCA205 pulmonary metastases eliminated tumors and 6 × 10^6 ^cells was the threshold dose for complete response whereas 2 × 10^6 ^cells were subtherapeutic. In addition, there was no response against the antigenically distinct MCA207 tumor. In an independent experiment, the dose of T cells required to completely eliminate 3-day pulmonary metastases was 2 × 10^6 ^indicating some inter-experimental variability in per-cell efficacy. Because of the critical role of CD4^+ ^T cells for therapy of s.c. or i.c. tumors it was not anticipated that the hyperexpanded CD8^+ ^cultures would mediate complete regression of tumors at these anatomic sites. Indeed, there was substantially less efficacy against 3-day s.c.tumors. Adoptive transfer of 4 × 10^7 ^cells showed a trend toward response (*P *= 0.061) with 1/5 mice cured in only one of two identically designed experiments (Figure 3C). In addition, a dose of 2 × 107 cells was subtherapeutic against 3-day i.c. tumors (Figure 3D).

Despite the rapid proliferation of CD8^+ ^T cells *in vitro*, there was no evidence of lymphoid hyperplasia when the mice were sacrificed to enumerate lung metastases 17 days after adoptive transfer. Moreover, there was no evidence of lymphoproliferative disease even when the hyperexpanded T cells were transferred into 5Gy TBI hosts bearing i.c. or s.c. tumors that had transient lymphodepletion of host cells. Thus, despite extensive proliferation *in vitro *the T cells did not demonstrate any evidence of transformation.

### Hyperexpanded CD4^+ ^T effector cells mediate regression of i.c. and s.c. tumors

**Figure 4:**
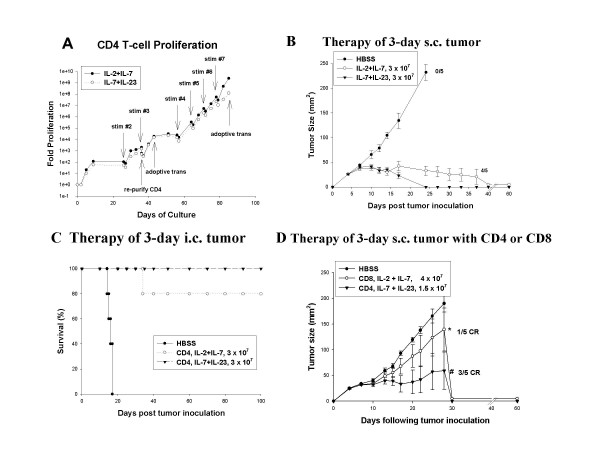
**Hyperexpanded CD4^+ ^T cells mediate regression of intracranial or subcutaneous tumors. **(**A) **CD62L^low ^TDLN cells were depleted of CD8^+ ^cells prior to anti-CD3 activation and were maintained in medium with IL-2 (4 U/ml) plus IL-7 (10 ng/ml) or alternatively with IL-7 (10 ng/ml) plus IL-23 (2 ng/ml) and were restimulated for 14 hrs with anti-CD3 mAb at the indicated time points. The total proliferation with indicated losses due to AICD or re-purification of CD4^+ ^cells is indicated. (B) Mice bearing 3-day s.c.tumors were treated with 5 Gy TBI followed by adoptive transfer of 3 × 10^7 ^CD4^+ ^T cells culture activated for 43 days and tumor size was measured. On day 37, one mouse from IL-2 + IL-7 group was euthanized due to progressive tumor growth, however complete regression was observed in the remaining 4 mice (P = 0.015 versus control). Complete regression was observed in all five recipients of IL-7 + IL-23 cultured CD4^+ ^T cells (P = 0.005 versus control). (**C**) Mice bearing 3-day intracranial tumors were treated with 5Gy TBI followed by adoptive transfer of 3 × 10^7 ^CD4^+ ^T cells culture activated for 43 days. Mice were followed for survival (P < 0.01 for both treatment groups versus control). (D) Mice bearing 3-day subcutaneous tumors were treated with 5 Gy TBI followed by adoptive transfer of 4 × 10^7 ^CD8^+ ^T cells hyperexpanded to greater than 10^8^-fold for 50 days, or 1.5 × 10^7 ^CD4^+ ^T cells hyperexpanded to greater than 10^8^-fold for 85 days. On day 28, 4 mice from CD8 treatment group (* P = 0.39 versus control) and 2 mice from CD4 treatment group (# P = 0.019 versus control) were euthanized due to progressive tumor growth but complete tumor regression was observed in the remaining mice.

The clinical utility of adoptive immunotherapy for patients with metastatic cancer is dependent on the ability of T cells to function at all anatomic sites of disease. To selectively activate CD4^+ ^T cells, the CD62L^low ^TDLN were depleted of CD8^+ ^cells with magnetic beads prior to anti-CD3 activation and again on day 36 of culture. The CD4^+ ^cells were activated with anti-CD3 mAb for 48 hrs and cultured in the presence of IL-7 and either IL-2 or IL-23 with anti-CD3 restimulation performed on days 26 and day 36 of culture. The rate of proliferation of CD4^+ ^cells was similar in the presence of IL-2 or IL-23 (Figure 4A). On day 43 of culture, cells cultured with IL-7 plus IL-2 were 87% CD4^+ ^and 11% CD8^+^, whereas cells cultured in IL-7 plus IL-23 were 98% CD4^+ ^and less than 1% CD8^+^. The T cells were adoptively transferred to hosts with 3-day s.c (Figure 4B) or 3-day i.c. (Figure 4C) tumors demonstrating that a dose of 3 × 10^7 ^cells cultured in the presence of either IL-2 or IL-23 was curative.

The CD4^+ ^T cells maintained in IL-7 plus IL-23 were subjected to continued repetitive anti-CD3 restimulation every 7 days starting on day 56. These conditions led to exclusive proliferation of CD4^+ ^T resulting in 1.2 × 10^8^-fold total proliferation by day 85 of culture. Despite extensive *in vitro *proliferation in response to antigen-independent stimulation for 85 days, 1.5 × 10^7 ^CD4 cells retained efficacy against 3-day s.c.tumors, with 3/5 mice achieving complete tumor regression (P = 0.019 versus control) (Figure 4D). As previously demonstrated, CD8^+ ^cells synchronously cultured in the presence of IL-2 plus IL-7 for 50 days and expanded to 10^8^-fold demonstrated minimal efficacy against subcutaneous tumors (P = 0.39 versus control) with 1/5 mice achieving complete tumor regression.

### Hyperexpanded cultures retain IFN-γ producing cells

**Figure 5:**
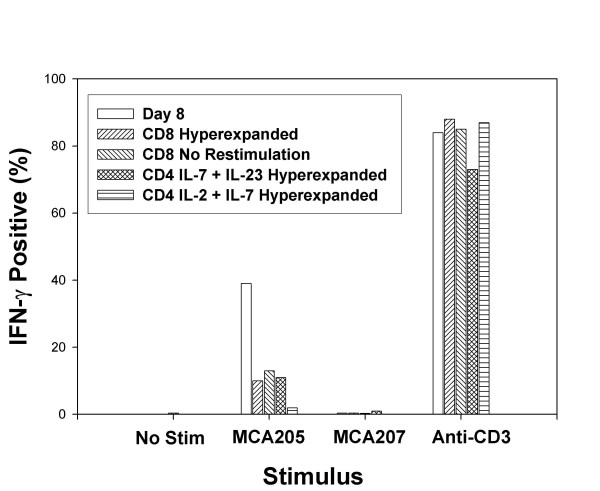
**Hyperexpanded CD4^+ ^and CD8^+ ^T cells produce IFN-γ in response to tumor stimulation. **CD62L^low ^TDLN cells were culture activated with anti-CD3 and IL-2 plus IL-7 for 23 days then were restimulated with anti-CD3 every 7 days. T cells were removed from culture on day 8, on day 36 for CD8^+ ^cultures, or day 43 for CD4^+ ^cultures. T cells were incubated without additional stimulus to determine spontaneous production of IFN-γ or with single cell digest of MCA205 or MCA207 tumors or with immobilized anti-CD3 mAb and Brefeldin A was added at 5 hrs and cells were harvested after 14 hrs. Intracellular IFN-γ was determined by FACS and the percentage of T cells is indicated.

The initial antigen priming event *in vivo *was driven by tumor-specific antigens but the segregation of the CD62L^low ^subset and all subsequent *in vitro *activation stimuli were antigen independent. Consequently, reactivity of cultures against tumor antigens might fluctuate over time. This possibility was analyzed by quantifying the percentage of culture activated T cells that produce IFN-γ specifically when exposed to tumor *in vitro*. For this assay, a single cell digest of *in vivo *propagated tumor is used which contains MHC class II^+ ^APC as well as tumor cells and is capable of stimulating both CD4^+ ^and CD8^+ ^T cells. As demonstrated in Figure 5, there was minimal spontaneous production of IFN-γ and minimal reactivity against the antigenically distinct MCA 207 tumor. By contrast, on day 8 of culture 39% of CD8^+ ^T cells produced IFN-γ in response to MCA 205 tumor cells. This percentage of IFN-γ positive CD8^+ ^cells decreased to 10% on day 36. CD8^+ ^T cells that were simply maintained in culture with IL-2 and IL-7 cytokine support but without anti-CD3 restimulation did not proliferate but remained viable. Under non-proliferative conditions, the percentage of MCA 205 reactive T cells was maintained at 13% on day 36. Similarly, CD4^+ ^T cells cultured in the presence of IL-7 plus IL-23 had 11% IFN-γ positive cells in response to MCA205 tumor with minimal spontaneous production and minimal response to MCA207 tumor digest. The *in vitro *assay of IFN-γ production may not be fully reflective of the *in vivo *capacity of T cells to mediate tumor regression because CD4^+ ^T cells cultured with IL-2 plus IL-7 had only 2% IFN-γ positive cells despite equivalent *in vivo *anti-tumor efficacy. The vast majority of T cells (73–86%) produced IFN-γ in response to anti-CD3 stimulation throughout the *in vitro *culture activation period.

## Discussion

These experiments demonstrate that T cells, sensitized to tumor antigens *in vivo*, can be activated *in vitro *under conditions that promote hyperexpansion of either the CD4^+ ^or CD8^+ ^subset while retaining their potent therapeutic efficacy against established tumors. A notable feature of *in vitro *activation is that it permits selection and enrichment of a minor subset of tumor-reactive precursor cells. The mechanism of antigen sensitization of T cells through cross-priming by APC within draining LNs provides a convenient localized anatomic source that is already highly enriched. When coupled with physical segregation based on phenotypic characteristics that distinguish between antigen-stimulated versus naïve T cells, enrichment to nearly 40% of tumor-specific T cells was achieved. One important aspect of this strategy for selection and enrichment is that it does not require pre-existing knowledge of the immunogenic tumor antigens and does not require freshly acquired T cells to exhibit effector function. These conditions have relevance for many clinical situations where tumor antigens are not yet described or where unique tumor antigens may be immunodominant. Moreover, signaling defects have been observed in freshly acquired T cells from tumor-bearing hosts that might impede segregation based on functional properties [42-44].

Starting with a highly enriched population of T cells we were able to use a powerful, yet antigen-independent, stimulus such as anti-CD3 mAb that preserved the initial TCR repertoire diversity. Interestingly, anti-CD28 stimulation was not required for this experimental model, presumably because the T cells had already received co-stimulation during APC-mediated priming *in vivo*. The principal advantage to *in vitro *activation is that the culture conditions can be adapted to optimize proliferation of distinct subsets of responding cells. It is important to note that anti-CD3 activation in the absence of exogenous cytokine support did not lead to T cell proliferation even among CD4^+ ^cells. The low cell density may have prevented secreted cytokines from reaching a critical threshold concentration. Moreover, IL-7, produced by non-hematopoetic cells and IL-23, produced by APC, mandated an exogenous source of these cytokines for *in vitro *culture activation. The combination of IL-2 and IL-7 provided rapid proliferation of CD8^+ ^T cells and preserved their viability after completion of the initial mitogenic burst. The reason this combination was effective is that IL-7 receptor α chain is constitutively expressed on naïve and memory T cells but is downregulated on activated T cells [45,46]. By contrast, the IL-2 receptor α chain is reciprocally expressed on activated cells in a transient manner. Thus, the combination of these two cytokines ensured continuous mitogenic signal transduction. IL-7 is crucial for development and homeostasis of T cells and is markedly increased following lymphodepletion. Therefore, there is considerable interest in employing lymphodepletion as a strategy to augment active as well as adoptive immunotherapy [47]. Likewise, exogenous IL-2 has been administered in the context of tumor antigen vaccination as well as in nearly every clinical application of adoptive transfer to provide helper function [48]. However, in addition to their mitogenic effects on antigen-stimulated T cells, systemic production of IL-7 or systemic administration of IL-2 has effects on irrelevant T lymphocytes, other hematopoetic cells, and the vasculature. The inability to target cytokine support specifically to the relevant T cells limits the effectiveness of *in vivo *cytokine administration. More importantly, we have clearly documented that adjunctive IL-2 inhibits trafficking of adoptively transferred T cells into intracranial or subcutaneous tumors [49]. By contrast, cytokine stimulation can be targeted specifically to effector cells under optimal conditions *in vitro *without adverse systemic effects on the host. Future experiments to adjust the sequence, and concentration of supplemental cytokines using more sophisticated schedules than employed here might provide superior effector function.

These experiments confirm the importance of CD4^+ ^T cells for therapy of tumors in certain anatomic sites, such as the brain and subcutaneous tissue. The slower rate of CD4^+ ^cell proliferation relative to CD8^+ ^cells following the initial anti-CD3 stimulation led to their rapid marginalization in mixed cultures. However, depletion of CD8^+ ^cells and use of cytokine combinations such as IL-7 and IL-23 favored the selective hyperexpansion of CD4^+ ^cells that retained potent *in vivo *function. IL-23 is a member of the IL-12 family of cytokines and contains the IL-12 p40 subunit that transduces signals through the shared IL-12β1 chain in addition to the unique p19 subunit [50,51]. The IL-23 receptor is expressed on memory but not naive CD4^+ ^cells, thus it is ideal for previously sensitized LN T cells. Myeloid cells, which are the natural source of IL-23, disappear rapidly in the *in vitro *cultures mandating an exogenous source. There is not a substantial amount of data on the effects of *in vivo *IL-23 administration, therefore its utility as a systemically administered adjuvant for T cell adoptive immunotherapy is unclear. However, the related cytokine IL-12 has substantial systemic effects that have limited its clinical use [52].

Although CD4^+ ^T cells have been investigated as a source of helper function for CD8^+ ^cytolytic cells our previous experiments have clearly established their stand-alone potential against MHC class II negative tumors [53]. CD4^+ ^T cell anti-tumor function is mediated through cross-presentation of specific tumor-antigens by tumor associated APC [54]. As demonstrated, CD4^+ ^cells cultured with IL-23 produced greater levels of INF-γ that would augment antigen presentation. Indeed, addition of tumor-reactive CD4^+ ^cells to tumor digest increases the reactivity of the CD8^+ ^cells. In addition to their autonomous effector functions, CD4^+ ^cells are required to generate a functional CD8^+ ^memory response *in vivo *[55,56]. Our recent experiments have demonstrated that adoptive transfer of effector T cells causes tumor destruction and sensitization of a secondary wave of regenerating host T cells [57]. In this regard, IL-23 stimulation of CD4^+ ^cells might be particularly useful because it, unlike IL-12, induces production of the pro-inflammatory cytokine IL-17 [58]. Our observation that such sensitization occurred even in hosts with partial tumor regression indicates that the presence of effector CD4^+ ^T cells and inflammatory conditions of tumor antigen acquisition by host APC are important in perpetuating the anti-tumor response.

Repetitive anti-CD3 stimulation was utilized to drive hyperproliferation of T cells yet the TCR/CD3 complex also activates genetic programs required for effector function. Effector molecules such as Fas, Perforin, and IFN-γ have autoinhibitory as well as paracrine inhibitory effects during culture activation. Viewed purely in operational terms, sequential *in vitro *activation first under conditions that optimize T cell proliferation, then with conditions that restore effector functions immediately before adoptive transfer, would be advantageous. Future experiments will explore whether it is possible to dissociate proliferative signaling pathways from those mediating effector function through selective transient gene inactivation. The quantitative aspects of hyperexpansion are of less scientific interest but do have some practical implications. The 10^8^-fold extent of proliferation far exceeded what was required to treat the tumor models employed. Moreover, the availability of uniformly primed T cells for mechanistic studies is not numerically limited when using an inbred strain. However these experiments establish an approach to maintain polyclonality and preserve effector function despite extensive antigen-independent proliferation. As such, the quantitative aspects of hyperexpansion may have relevance to certain clinical situations where an autologous source of antigen-primed T cells may be limited and extensive host tumor burden may demand a large number of effector T cells.

## Supplementary Material

Additional File 1 This is a table describing the percentage of cells expressing various TCR Vβ family members at three different time points. Click here for file
