# Supplementary material for: Adoptive immunotherapy of cancer with polyclonal, 108-fold hyperexpanded, CD4+ and CD8+ T cells
Source: J Transl Med. 2004 Nov 26;2:41. doi: 10.1186/1479-5876-2-41 (PMC535812; doi:10.1186/1479-5876-2-41)
Supplement: Additional File 1 — This is a table describing the percentage of cells expressing various TCR Vβ family members at three different time points. [file 1479-5876-2-41-S1.doc]

# Table 1. TCR Vβ Phenotype

|  | Day 0 | | | | Day 22 or Day 23 | | | Day 50 | | |
| --- | --- | --- | --- | --- | --- | --- | --- | --- | --- | --- |
|  | Exp.1 | | Exp.2 | | Exp.1 | Exp.2 | Exp.3 | Exp.1 | Exp.2 | Exp.3 |
|  | TDLN  TCR+ | CD62L^loww^  TCR+ | TDLN  TCR+ | CD62L^lo^  TCR+ | IL-2/7 | IL-2/7 | IL-2/7 | IL-2/7 | IL-2/7 | IL-2/7 |
| Vβ 2 | 5.2 | 5.9 | 5.3 | 8.7 | 3.2 | 3.1 | 3.1 | 2.3 | 1.8 | 1.4 |
| Vβ 3 | 2.7 | 5.0 | 2.6 | 6.3 | 0.3 | 1.6 | 2.3 | 0.7 | 0.9 | 0.24 |
| Vβ 4 | 4.5 | 6.8 | 4.5 | 7.8 | 1.8 | 2.9 | 2.8 | 5.1 | 1.7 | 1.8 |
| Vβ 5.1/5.2 | 6.9 | 6.6 | 6.8 | 6.9 | 17.6 | 17.4 | 18.1 | 15.3 | 10.4 | 17.3 |
| Vβ 6 | 5.2 | 8.6 | 6.1 | 8.3 | 6.6 | 8.6 | 8.1 | 5.8 | 4.6 | 5.7 |
| Vβ 7 | 3.3 | 5.5 | 4.3 | 6.7 | 4.7 | 3.2 | 4.1 | 1.7 | 15.5 | 4.0 |
| Vβ 8.1/8.2 | 10.8 | 15.2 | 12.1 | 17.8 | 13.0 | 13.4 | 13.4 | 21 | 6.0 | 12.9 |
| Vβ 8.3 | 4.9 | 7.5 | 4.2 | 6.8 | 8.0 | 7.9 | 8.6 | 4.7 | 5.4 | 3.3 |
| Vβ 9 | 1.5 | 1.8 | 1.2 | 2.5 | 1.9 | 1.5 | 1.6 | 10.5 | 0.8 | 0.5 |
| Vβ 10 | 3.3 | 4.1 | 3.8 | 4.2 | 3.9 | 4.9 | 5.3 | 3.6 | 2.2 | 6.2 |
| Vβ 11 | 4.4 | 3.9 | 4.4 | 4.8 | 7.6 | 10.2 | 6.2 | 4.6 | 6.7 | 9.1 |
| Vβ 12 | 2.3 | 3.4 | 2.3 | 3.8 | 2.3 | 2.2 | 2.5 | 2.2 | 7.7 | 1.7 |
| Vβ 13 | 2.1 | 4.1 | 2.6 | 4.8 | 3.5 | 5.9 | 2.9 | 7.4 | 3.7 | 4.7 |
| Vβ 14 | 3.6 | 6.1 | 4.5 | 6.7 | 1.9 | 2.3 | 2.7 | 0.65 | 0.6 | 1.1 |
| Vβ 17 | 0.33 | 1.2 | 0.5 | 1.3 | 0.12 | 0.3 | 0.03 | 0.12 | 0.08 | 0.03 |
